# Contributions of Sensory Coding and Attentional Control to Individual Differences in Performance in Spatial Auditory Selective Attention Tasks

**DOI:** 10.3389/fnhum.2016.00530

**Published:** 2016-10-20

**Authors:** Lengshi Dai, Barbara G. Shinn-Cunningham

**Affiliations:** Department of Biomedical Engineering, Boston UniversityBoston, MA, USA

**Keywords:** selective attention, subcortical, cortical, individual differences, event-related potential, envelope following response, simultaneous measurement

## Abstract

Listeners with normal hearing thresholds (NHTs) differ in their ability to steer attention to whatever sound source is important. This ability depends on top-down executive control, which modulates the sensory representation of sound in the cortex. Yet, this sensory representation also depends on the coding fidelity of the peripheral auditory system. Both of these factors may thus contribute to the individual differences in performance. We designed a selective auditory attention paradigm in which we could simultaneously measure envelope following responses (EFRs, reflecting peripheral coding), onset event-related potentials (ERPs) from the scalp (reflecting cortical responses to sound) and behavioral scores. We performed two experiments that varied stimulus conditions to alter the degree to which performance might be limited due to fine stimulus details vs. due to control of attentional focus. Consistent with past work, in both experiments we find that attention strongly modulates cortical ERPs. Importantly, in Experiment I, where coding fidelity limits the task, individual behavioral performance correlates with subcortical coding strength (derived by computing how the EFR is degraded for fully masked tones compared to partially masked tones); however, in this experiment, the effects of attention on cortical ERPs were unrelated to individual subject performance. In contrast, in Experiment II, where sensory cues for segregation are robust (and thus less of a limiting factor on task performance), inter-subject behavioral differences correlate with subcortical coding strength. In addition, after factoring out the influence of subcortical coding strength, behavioral differences are also correlated with the strength of attentional modulation of ERPs. These results support the hypothesis that behavioral abilities amongst listeners with NHTs can arise due to both subcortical coding differences and differences in attentional control, depending on stimulus characteristics and task demands.

## Introduction

A number of recent studies suggest that listeners with normal hearing thresholds (NHTs) may suffer from auditory neuropathy, or a loss of ascending auditory nerve fibers (Schaette and McAlpine, 2011; Plack et al., 2014; Bharadwaj et al., 2015). This kind of loss appears to have a particularly strong impact on how well listeners can understand speech in noise or when there are competing sources (Hind et al., 2011; Ruggles and Shinn-Cunningham, 2011; Ruggles et al., 2012). Recent work in animal models are consistent with these reports, showing that auditory neuropathy can be fairly severe without impacting the quietest sound that can be detected (e.g., see Kujawa and Liberman, 2009; Lin et al., 2011; Lobarinas et al., 2013). Evidence suggests that low-spontaneous rate auditory nerve fibers, which only become active at supra-threshold levels, are more susceptible to damage from noise exposure than high-spontaneous rate fibers, which respond at hearing threshold (Furman et al., 2013); this helps explain why supra-threshold sound perception is degraded even though detection thresholds are unaffected. Given this, auditory neuropathy, very like driven by noise exposure and aging (Schaette and McAlpine, 2011; Bharadwaj et al., 2014, 2015; Plack et al., 2014), is a likely contributor to individual differences in the encoding of subtle spectro-temporal features of supra-threshold sound. Such features are critical for segregating sound sources; if a listener cannot segregate sources, then they will have trouble directing attention to whichever source is of interest. Given this, auditory neuropathy may explain why some NHT listeners experience communication problems in noisy environments (Shinn-Cunningham et al., in press).

Consistent with this, in one recent set of studies, NHT subjects were asked to report spoken digits from straight ahead while ignoring otherwise identical digits ±15^°^ off center. Despite having normal auditory thresholds, performance varied from below 40% to nearly 90%; moreover, almost all mistakes arose because listeners reported the content of one of the competing streams, rather than because they failed to understand the digits in the mixture (Ruggles and Shinn-Cunningham, 2011; Ruggles et al., 2011). Importantly, these difficulties in focusing on target speech amidst competing speech were correlated with the strength of the subcortical response to periodic sound, known variously as the frequency-following response (FFR) or the envelope following response (EFR; see Ruggles and Shinn-Cunningham, 2011; Ruggles et al., 2012). These results suggest that poor subcortical encoding can lead to deficits in the ability to focus selective auditory attention on a source from a particular direction.

Still, it is clear that individual differences in the ability of listeners to understand speech in noisy settings are not always due to differences in sensory coding fidelity; everything from general cognitive ability to aging affects the ability to understand speech in complex settings (e.g., see Gordon-Salant et al., 2006, 2007; Singh et al., 2008, 2013; Grose et al., 2009; Grose and Mamo, 2010, 2012; Nakamura and Gordon-Salant, 2011; Rönnberg et al., 2011; Weisz et al., 2011; Banh et al., 2012; Benichov et al., 2012; Hall et al., 2012; Noble et al., 2012; Tun et al., 2012; Anderson et al., 2013; Brungart et al., 2013; Veneman et al., 2013). Consistent with this, most of the studies demonstrating a link between sensory coding deficits and failures of selective auditory attention were designed so that the features that distinguished the target source from competing speech streams differed only modestly (e.g., only 15^°^ of separation between competing streams; see Ruggles et al., 2011, 2012), on the edge of what even “good” listeners are able to use reliably. By design, performance in such paradigms depends on subtle differences in the robustness of temporal coding of supra-threshold, audible sound. These subtle differences are likely the primary limitation on performance in these experiments, and thus correlate with individual differences in ability, even though more central differences in processing ability may also be present.

Selective auditory attention engages multiple regions that must work together to modulate the sensory representation of sound based on task demands (e.g., see Giesbrecht et al., 2003; Fritz et al., 2007; Hill and Miller, 2010). In situations where streams are easy to segregate and have perceptually distinct features, differences in the efficacy of these cortical control networks are likely to determine individual performance and perceptual ability. Indeed, one recent study shows that there are large inter-subject differences in how well listeners can identify melody contours when there are competing melodies from widely separated directions, a task in which segregation and selection probably does not depend on individual differences in sensory coding (Choi et al., 2014). Yet, in this study, individual differences in performance were consistent across conditions, and performance correlated with how strongly cortical responses to the competing melodies were modulated by attentional focus (Choi et al., 2014). These results suggest that in addition to differences in subcortical coding fidelity, there are significant, relatively central individual differences in the ability to control selective auditory attention, and that these consistent individual differences determine behavioral ability on tasks where peripheral coding does not limit performance.

Still, the relationship between sensory coding differences and differences in cortical control of attention are not entirely clear. For instance, it is possible that differences in the strength of attentional modulation arise not from differences in central control, but are driven instead by differences in sensory coding fidelity. For instance, if coding fidelity is so poor that a listener cannot separate the target source from competing sources, it will necessarily lead to failures in suppressing neural responses to competing sound sources (Shinn-Cunningham and Best, 2008; Shinn-Cunningham and Wang, 2008). It is possible that this kind of cascade could explain the individual differences observed by Choi et al. (2014). Specifically, they did not measure subcortical coding fidelity in their subjects; it is possible that listeners who performed well and were able to modulate cortical auditory responses strongly were the listeners with the most robust peripheral encoding of supra-threshold sound. As a result, these listeners may have been best at segregating the competing melodies and suppressing the unimportant streams in the mixture.

The current study was designed to test directly whether individual differences in sensory coding and differences in the central control of attention both contribute to the ability to analyze one target sound stream when it is presented with simultaneous, competing streams. We undertook two experiments to examine the relationships between subcortical sensory coding fidelity, the strength of attentional modulation of cortical responses and behavior performance. In both experiments, we measured all three (subcortical coding, attentional modulation and performance) in the same listeners at the same time. By varying stimulus characteristics, we expected to shift the balance in how important peripheral and central factors were in determining performance, allowing us to demonstrate that these factors interact to affect the ability to perform spatial auditory attention tasks.

Both subcortical and cortical responses can be measured using electroencephalography (EEG). However, the experimental design typically depends on which kind of response a study aims to measure; the type of stimuli, timing of the stimuli, number of stimulus repetitions, EEG sampling rate, electrode configuration and EEG data pre-processing and processing schemes (to name some of the experimental parameters) usually are set differently depending on which kind of measure is desired. Perhaps as a result, few studies have simultaneously measured subcortical and cortical responses. Still, if an experiment is designed with both subcortical and cortical response characteristics in mind, they can be measured in the same experiment, at the same time (e.g., see Hackley et al., 1990; Krishnan et al., 2012). In the current study, we measured cortical and subcortical responses during selective auditory attention tasks in order to examine individual differences in performance and how they relate to both measures. By recording subcortical and cortical data at the same time in our subjects, we guaranteed that the same physiological and psychological conditions were at play in each measurement, allowing us to compare outcomes directly.

To measure cortical responses, we considered auditory event-related potentials (ERPs), which are elicited by auditory events such as onsets of notes in a melody or syllables in an ongoing stream of speech. By comparing the magnitude of ERPs to the same mixture of auditory inputs when listeners attend to one stream vs. when they attend to a different stream, we can quantify the degree of top-down control of selective attention for individual listeners (e.g., see Choi et al., 2014).

We analyzed subcortical responses using the EFR, a measure that quantifies the degree to which the subcortical portions of the pathway phase lock to ongoing temporal periodicities in an input acoustic stimulus (Zhu et al., 2013). By focusing on relatively high-frequency modulation (above 100 Hz), the brainstem response, rather than cortical activity, dominates this measure (see Shinn-Cunningham et al., in press). In addition, a number of past studies have related EFRs to perceptual ability (Krishnan et al., 2009; Bidelman et al., 2011; Carcagno and Plack, 2011; Gockel et al., 2011).

The two different experiments were similar, but we hypothesized that they would yield different results. In both experiments, there were two potential target streams, one from the left and one from the right of the listener. From trial to trial, we randomly varied which stream was the target, using a visual cue to indicate whether the listener should direct attention to the stream on the left or the stream on the right. While the overall structure of the two experiments was grossly similar, the tasks and auditory stimuli differed in order to try to isolate different factors contributing to individual differences.

Experiment I presented listeners with two streams of repeated complex tones and asked listeners to count pitch deviants in the attended stream. Because the pitch deviations were small, we hypothesized that subject differences in the ability to report the correct number of deviants would be related to differences in subcortical temporal coding. In Experiment I, the spatial separation of the two streams was large. Therefore, we did not expect differences in subcortical temporal coding to limit how well or fully listeners could focus spatial attention on the target stream. While we expected subjects to differ from one another in the degree to which they could focus spatial attention and modulate cortical responses to the competing streams, we did not expect these subject differences in cortical control to correlate with either behavioral performance or with the subcortical coding fidelity given how clearly segregated we expected the competing streams to be.

Experiment II presented listeners with two potential target streams that each comprised simple melody contours. Listeners were asked to report the shape of the melody of the attended stream, which consisted of sequences of high and low pitches separated by a small pitch difference. Thus, as in Experiment I, the task required listeners to judge small pitch variations within an ongoing stream. In contrast to Experiment I, we made the ability to selectively focus attention on the target stream challenging by including a third, distractor stream melody from straight ahead and by reducing the spatial separation between the competing streams. As a result, the ability to selectively focus attention was more of a bottleneck in Experiment II than in Experiment I. We hypothesized that in Experiment II, performance would depend on individual differences in subcortical temporal coding, because coding fidelity would determine both how well listeners could hear the melody contour and how well they could use the modest spatial differences that differentiated the target stream from the two competing streams. We further hypothesized that individual differences in the strength of subcortical coding would partially correlate with both the degree of cortical modulation of ERPs and with performance on the selective attention task. However, we also hypothesized that even after factoring out correlations with subcortical responses, remaining differences in performance would correlate with attentional modulation strength. This final result would suggest that in Experiment II, central differences in attentional control differed across listeners and directly impacted individual differences in the ability to perform the task, even after accounting for the effects of sensory coding fidelity.

## Common Methods

### Subjects

All subjects were screened to confirm that they had NHTs at frequencies between 250 Hz and 8000 Hz (thresholds of at most 20 dB HL) for both ears. This study was carried out in accordance with the recommendations of the Boston University Charles River Campus Institutional Review Board (CRC IRB). All subjects gave written informed consent in accordance with the Declaration of Helsinki. The protocol was approved by the CRC IRB. All subjects were compensated at the rate of $25 per hour, and were paid a $0.02 bonus for each correct response to ensure that they remained attentive throughout the task.

### Equipment

Subjects sat in a sound-treated booth while performing the tasks using a PC keyboard and monitor. The PC controlled the experiment using Psychtoolbox 3 (Brainard, 1997) and Matlab (Mathworks; Natick, MA, USA). The control code also generated triggers that were recorded to mark the times of key events. Auditory stimuli were presented through a TDT System Three unit (Tucker-Davis Technologies, Alachua, FL, USA) and ER-1 insert headphones (Etymotic, Elk Grove Village, IL, USA).

A BioSemi Active Two System (Amsterdam, Netherlands) recorded EEG signals using a 4.096 kHz sampling rate. Recordings were taken from 32 active scalp electrodes in the standard 10/20 configuration. Two additional electrodes were placed on the mastoids; during analysis, the EEG recordings were re-referenced to the mean of the two mastoid electrodes. Synchronized triggers from the TDT system were recorded simultaneously with the EEG data, which were stored on the controlling PC.

### Stimuli

Stimuli were generated in Matlab (Mathworks; Natick, MA, USA) using a sampling rate of 48.828 kHz. Each trial consisted of a mixture of simultaneous, isochronous sequences of complex tones that had different repetition rates, so that onsets of the notes in the different sequences were resolvable in time. In both experiments, two of these streams were potential target streams (Stream A and Stream B). On each trial, we varied the perceived laterality of the streams using interaural time differences (ITDs), so that one of the potential target streams was heard from one hemifield, and the other potential target from the opposite hemifield (chosen randomly from trial to trial). Experiment I presented only two streams, while Experiment II included a third, central stream that was never the focus of attention (Stream C). Each of the complex tones in both Stream A and Stream B consisted of the first 33 harmonics of some fundamental frequency, all of equal amplitude, added in sine phase. In Experiment II, the notes in Stream C were made up of the first three harmonics of their fundamentals, all of equal amplitude, added in sine phase. All notes in both experiments were played at a level of 70 dB SPL (root-mean-squared).

The fundamental frequencies of the notes in each stream as well as the repetition rates of the notes were carefully chosen to ensure that they were not harmonically related to each other or to 60 Hz. Because of this design, when we binned responses to notes in each stream, any interference from neural responses to competing streams and any ongoing line noise was random across bins, and tended to cancel out. The temporal structure of the trials permitted us to analyze cortical EEG responses to note onsets in each stream by examining responses at the correct time points; as a consequence of this design, the number of notes in Stream A and Stream B differed (For Stream A and Stream B, respectively, the number of notes was 10 and 8 in Experiment I and 5 and 4 in Experiment II; Stream C in Experiment II had 4 notes). In order to extract the brainstem EFRs from the EEG, the stimuli in half of the trials in each experiment were presented in negative polarity (see Skoe and Kraus, 2010).

### Task Design

**Figure 1:**
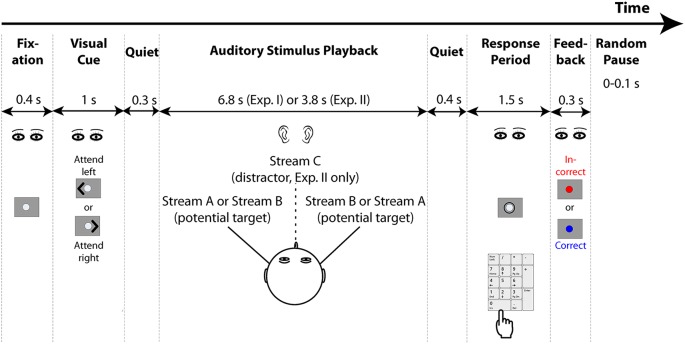
**General structure of the experiments.** Both experiments start with the presentation of a fixation dot on the screen, after which a visual cue appears to indicate the direction to which listeners should attend. Two potential target streams are presented symmetrically in the left and right hemispheres using interaural time differences (ITDs; Experiment II also presents a distractor stream from the center, which is always ignored). Listeners have a brief response period after the conclusion of the auditory stimuli in which to respond using a computer keypad. Feedback is then provided to tell them whether or not their response was correct. After a random pause, the next trial begins.

The general task structure is shown in Figure 1. Each trial started with the presentation of a 0.4-s long fixation dot, followed by a 1-s long visual cue. The cue was an arrowhead that appeared to one side of the fixation dot and pointed either to the left or right, indicating the direction of the target stream on that trial (selected randomly for each trial, separately for each subject). After the cue ended, there was a 0.3 s of pre-stimulus quiet period, then the auditory stimulus began (6.8 s of duration for Experiment I and 3.8 s of duration for Experiment II). A 0.4 s of post-stimulus silent period followed the auditory stimulus presented on each trial, after which a circle appeared around the fixation dot to indicate the response period, which lasted 1.5 s. Listeners were instructed to maintain gaze to the fixation dot/cue, and then, during the response period, to use number keys on the computer keyboard to provide their response. The program recorded the last button push within the response period as the registered answer, so subjects could correct a mistaken button push if they changed their answer within that time (if there was no response during the response period, no response was recorded and the trial was counted as incorrect). Feedback was given after the response period ended: the fixation dot flashed for 0.3 s, either red for an incorrect response or blue for a correct response. After the end of the visual feedback, the next trial began after a random pause (0–0.1 s, randomly selected on each trial from a uniform distribution).

### Cortical ERP Analysis

To isolate cortical responses from the scalp-recorded EEG, signals were band-pass filtered from 2 to 25 Hz using the *eegfiltfft*.*m* function in EEGLab toolbox (Delorme and Makeig, 2004). We focused our cortical analysis of auditory ERPs on channel Cz (channel 32 in the 10/20 system), where they tend to be greatest. For each trial, we analyzed epochs of the EEG from −0.2 s (before the sound stimulus began) to the end of the stimulus. For each such epoch, we found the maximum absolute peak voltage. In order to reduce contamination from movement and other artifacts, for each subject we created a histogram of peak values across trials, and then rejected trials in the top 15% of each subject’s distribution from further analysis.

Using the remaining trials, we used a bootstrap procedure to compute average ERPs to the onsets of notes in Stream A and Stream B separately for when Stream A was the target and when Stream B was the target. Specifically, for each attention condition for each subject, we used a 200-draw bootstrap procedure with replacement (100 trials per draw). The N1 magnitude of each note onset ERP was taken to be the local minimum between 100–220 ms after the onset of the corresponding note. The P1 magnitude of each ERP was taken to be the local maximum in the period from 30–100 ms after the note onset. We computed the difference in these magnitudes to estimate the average peak-to-peak P1-N1 magnitude. Thus, for each subject, we estimated the P1-N1 magnitude in response to each note onset in Stream A and Stream B when Stream A was the target and separately when Stream B was the target. We denote these magnitudes as $M_{\text{s},\text{n}}^{\text{focus}}$, where *s* is the stream containing the note onset being analyzed (either A or B), *focus* denotes whether that stream was *Attended* or *Ignored*, and n denotes the temporal position of the note in the corresponding stream.

Since it takes time for an auditory stream to be perceptually segregated from a sound mixture (Cusack et al., 2004; Best et al., 2008), we quantified the strength of top-down attentional modulation for each individual note in each stream. However, because the effects of endogenous attention and attention switching could interfere with top-down modulation of responses to the first notes in each stream, we omitted these from analysis of the effects of top-down attention on the P1-N1 magnitude.

Top-down executive control is expected to modulate the sensory representation of sound in the cortex, leading to reduced responses when a stream is ignored compared to when it is attended, which may be due to both suppression of the stream when it is ignored and enhancements of the stream when it is attended (e.g., see Picton and Hillyard, 1974; Choi et al., 2014). Given this, we expected $M_{\text{s},\text{n}}^{\text{Attended}}$ to be larger than $M_{\text{s},\text{n}}^{\text{Ignored}}$. However, ERP magnitudes vary significantly across subject, due to differences in brain geometry, electrode impedance, and other “nuisance” factors; these factors cause shifts in measured ERPs that are constant on a logarithmic scale. Computing differences in ERP amplitudes on a linear scale would not compensate for these changes in overall strength. Consistent with this, past experiments in our lab suggest that the percentage change in ERP amplitudes, or (equivalently in a mathematical sense) the difference of the ERP amplitudes on a logarithmic scale, is a good way to quantify individual differences in how strongly attention modulated responses, as if the effect of attention is well modeled as a multiplicative gain change in response amplitude (e.g., Choi et al., 2014). Therefore, to quantify an individual’s ability to modulate the neural representation based on top-down attention, we computed the Attentional Modulation Index (AMI) for each stream by computing the difference of the log of the magnitudes of $M_{\text{s},\text{n}}^{\text{Attended}}$ and $M_{\text{s},\text{n}}^{\text{Ignored}}$. Specifically, the AMI was computed for each subject as the average across note onsets (from the second to final note) of the log of the ratio of $M_{\text{s},\text{n}}^{\text{Attended}}$ over $M_{\text{s},\text{n}}^{\text{Ignored}}$:

(1) $${\text{AMI}}_{\text{s}}\;=\;\frac{1}{N-1}\sum_{n\;=\;2}^{N}\text{log}\left(\frac{M_{\text{s},\text{n}}^{\text{Attended}}}{M_{\text{s},\text{n}}^{\text{Ignored}}}\right)$$

where *N* is the number of notes comprising stream *s* (in Experiment I, *N* = 10 for Stream A and *N* = 8 for Stream B; in Experiment II, *N* = 5 for Stream A and *N* = 4 for Stream B). Defined this way, the AMI should be zero if attention has no effect on the neural representation of the stream ($M_{\text{s},\text{n}}^{\text{Attended}}$ equals $M_{\text{s},\text{n}}^{\text{Ignored}}$) and increases monotonically with the strength of attentional modulation of the neural responses.

### EFR Analysis

To isolate subcortical responses from the scalp-recorded EEG, signals were high-pass filtered with a 65 Hz cutoff using the *eegfiltfft.m* function in EEGLab toolbox (Delorme and Makeig, 2004). We then quantified the fidelity with which the subcortical response of each subject encoded the fundamental frequency of identical complex tones in the presented streams (Bharadwaj and Shinn-Cunningham, 2014).

For each complex tone that we analyzed, we treated each identical tone repetition as an independent sample, regardless of its temporal position in a stream. For each of these repetitions, we analyzed the epoch from the note onset to the end of the note. We combined an equal number of positive polarity and negative polarity repetitions to compute the EFR (Skoe and Kraus, 2010; Shinn-Cunningham et al., in press). In order to achieve the best possible signal-to-noise ratio (SNR) in our estimates, we combined measurements across the EEG sensors using complex principal components analysis (Bharadwaj and Shinn-Cunningham, 2014). We quantified the EFR using the phase locking value (PLV; see Lachaux et al., 1999), a normalized index that ranges from 0 (no phase locking across trials) to 1 (perfect phase locking). Importantly, the number of repetitions used in this analysis determines the noise floor of the PLV, making it easy to interpret the results (Zhu et al., 2013).

Past work shows that selective auditory attention has a negligible effect on the EFRs generated by subcortical structures (Varghese et al., 2015). In Experiment I, we tested this by comparing PLVs in response to the most commonly repeated notes in each stream when that stream was attended vs. when it was ignored. For this analysis, we used a 200-draw bootstrap procedure with replacement (400 repetitions per polarity per draw) separately when listeners attended to the stream the notes were in and when listeners ignored the stream they were in. In Experiment II, we reduced the number of notes per stream and had fewer repetitions of the same notes per trial. Because of this, there were not enough trials to allow a direct comparison of EFRs to the same notes when listeners attended to the stream they were in vs. when that stream was ignored in Experiment II.

In both experiments, we quantified the strength of the EFRs for individual subjects by combining all repetitions of the most commonly repeated notes in Stream A and in Stream B, collapsing across conditions when the stream containing the notes was attended and when it was ignored. We used a 200-draw bootstrapping with replacement, with 500 repetitions per polarity per draw.

Like cortical ERPs, individual differences in the absolute EFR are influenced by various nuisance factors (e.g., brain geometry, electrode impedance, overall cortical noise levels; Bharadwaj et al., 2014, 2015). Within an experimental session for a given subject, these factors should affect both Stream A and Stream B EFRs identically on a logarithmic scale. Thus, we planned to quantify individual differences in subcortical coding using “normalized” EFR measures computed as the ratio of the EFR to Stream B notes to the ratio in Stream A notes, thereby canceling out nuisance factors (see also Bharadwaj et al., 2015, which demonstrates that individual differences in subcortical coding fidelity are better described by normalized EFRs than by absolute EFR strength).

Mid- to high-frequency stimulus content is the dominant signal driving EFRs (Zhu et al., 2013). As described in detail below, in both Experiment I and Experiment II, all of the harmonics but the fundamental in Stream A overlapped with the lower half of the spectral content of the notes in Stream B. However, the upper half of the spectrum of the notes in Stream B did not overlap with any other stimulus components. Because of this design, we expected the EFR in response to the notes of Stream B to be relatively strong for all subjects; the mid- to high-frequencies in the Stream B notes were not masked and also had deep modulations to drive the EFR (see Bharadwaj et al., 2014, 2015). In contrast, because Stream A notes were spectrally masked due to the interfering spectral content of Stream B (and thus had reduced modulation depth in the mid- and high-frequency portions of the stimulus), we expected these EFRs to depend more directly on the degree of cochlear neuropathy in an individual subject (Bharadwaj et al., 2014, 2015). Therefore, the ratio of the PLV to notes in Stream B divided by the PLV to notes in Stream A should be relatively small in good listeners (strong EFR to Stream B notes divided by a relatively strong EFR to Stream A notes) and large in listeners with a reduced number of auditory nerve fibers (strong EFR to Stream B notes divided by a relatively weak EFR to Stream A notes). By this logic, we expected this ratio to be negatively correlated with differences in how well listeners could perform the behavioral task, which relied, in both tasks, on the ability to discern small pitch differences between notes in the attended stream. To quantify these individual differences, we thus computed the PLV ratio for each subject as:

(2) $$PLVR_{\text{s}}\;=\;\frac{PLV_{\text{StreamB},\text{s}}}{PLV_{\text{StreamA},\text{s}}}$$

where *PLV_Stream x,s_* is the PLV of the EFR to the repeated notes in Stream x for subject *s*.

### Statistical Tests

Experimental factors were analyzed using multi-way ANOVAs based on mixed-effects models (Baayen, 2008) implemented in R (Foundation for Statistical Computing). Subject-related factors, which were not assumed to comply with homoscedasticity, were treated as random effects. All other factors and interactions were treated as fixed-effect terms (although some factors were nested, precluding inclusion of some interaction terms). To prevent over-fitting and determine the most parsimonious model, we compared models with and without each random effect term using the Akaike information criterion (AIC; Pinheiro and Bates, 2006). All data sets were checked for normality using the Kolmogorov–Smirnov test.

In addition, we examined individual differences by looking for correlations between variables. Significance was tested by computing the Pearson correlation coefficient; *p*-values were then computed using a two-tailed student’s *t* test.

## Experiment I

Experiment I presented listeners with two ongoing tone streams with different spectral content, and asked listeners to count pitch deviants in the attended stream. We measured the strength with which the subcortical EFR phase locked to the pitch of notes making up each stream, and the strength of cortical responses to the onsets of the notes in each stream.

### Materials and Methods

#### Subjects

Eleven subjects (8 males, 3 females, aged 21–41) were recruited.

#### Stimuli

**Figure 2:**
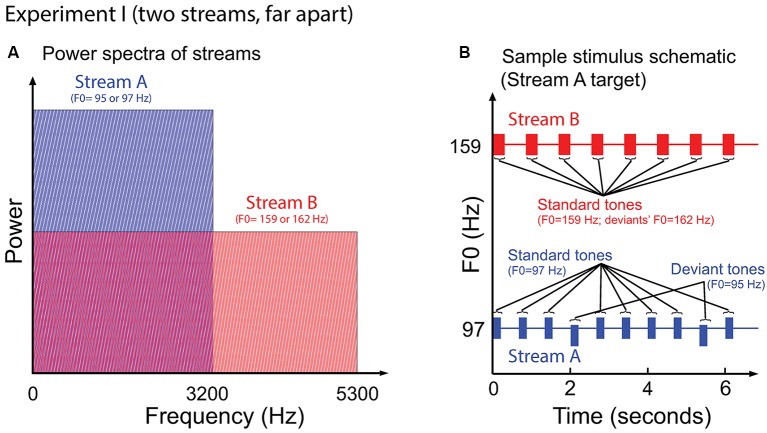
**(A)** Power spectrum of the auditory streams in Experiment I. Tones in Stream A were wholly masked by tones in Stream B, but not vice versa. **(B)** Sample schematic of auditory stimuli in Experiment I. “Stream A,” which is the target, has two “deviant” tones, while “Stream B” presents only standard tones. Listeners must report the number of deviant tones in the target.

Figure 2 illustrates the auditory stimuli used in Experiment I. Stream A was made up of 10 tones of 200 ms duration, separated by an inter-stimulus (onset to onset) interval (ISI) of 668 ms. Stream B was made up of eight complex tones of 300 ms duration, separated by an ISI of 849 ms. Most of the notes of each stream had the same fundamental frequency, of either 97 Hz (Stream A) or 159 Hz (Stream B); however, occasional “deviant” notes had a fundamental frequency that differed from the “standard” notes by 0.3 semitones (95 Hz and 162 Hz for Stream A and Stream B, respectively). All tones were time windowed with cosine-squared onset and offset ramps to reduce spectral splatter (10-ms duration). With this design, the spectral content of Stream A (97–3201 Hz) overlapped with the lower end of the spectrum of Stream B (159–5247 Hz); however, the content of Stream A did not interfere with the representation of the upper harmonics of the notes in Stream B (see Figure 2A).

Stream A and Stream B were simulated from different hemifields using ITDs of ±676 μs, chosen randomly on each trial. The distractor stream (chosen randomly on each trial) only contained standard tones. On any given trial, the target stream was randomly set to have 0, 1, or 2 deviants, with likelihoods of 50%, 35% and 15%, respectively. The deviant notes in the target stream were chosen randomly, with the constraint that the first note was always a standard, a fact of which the subjects were aware. The pitch difference between standard and deviant tones (0.3 semitones) is close to the difference limen (Moore and Peters, 1992), making the task challenging and encouraging listeners to focus attention on the target stream to perform the task well.

#### Task Design

The experiment was organized into blocks of 40 trials. The first block was a training session (data not included in later analysis), followed by seven test blocks (280 trials). Within each block, Stream A was the target stream for 20 trials and Stream B was the target stream in the other 20 trials. The trials were presented in a different random order for each subject.

During the response period (when a circle appeared around the fixation dot), listeners were asked to report the number of deviants in the target stream (0, 1, or 2) using the numbers on a computer keyboard. Feedback indicated whether the reported count was correct (fixation dot flashed to blue) or not (fixation dot flashed to red).

### Data Analyses

#### Behavior

Percent correct responses were calculated separately when the target stream was Stream A and when it was Stream B. These values were computed independently for each subject, averaging across trials in the seven test blocks.

#### ERP Measurement

We computed $M_{\text{s},\text{n}}^{\text{Attended}}$ and $M_{\text{s},\text{n}}^{\text{Ignored}}$ for notes 2–10 (Stream A) and notes 2–8 (Stream B) for each subject. We averaged these magnitudes over notes to summarize the strength of the attentional modulation for each subject (*AMI_s_*).

#### Subcortical Measurement

In each trial, we obtained responses to a minimum of eight identical standard tones from Stream A and six identical standard tones from Stream B; the number on a given trial depended on which stream contained deviants and how many deviants were present on that trial. For instance, in the example shown in Figure 2, where Stream A was the target and contained two deviants, the trial generated eight standard-tone repetitions for Stream A and eight standard-tone repetitions for Stream B. Thus, across all trials, there were at least 1120 (8 × 140) repetitions of the standard from Stream A in both attention conditions (when Stream A was attended and when Stream B was attended). Similarly, there were at least 840 (6 × 140) repetitions of the standard from Stream B in the two attention conditions.

### Results

#### Behavior

The mean percentage of correct responses was 76.49% when listeners attended to Stream A (range: 50.00–97.14%; standard deviation: 16.21%) and 85.47% when they attended to Stream B (range: 68.75–99.29%; standard deviation: 11.02%). Subjects were significantly better at the task when attending to Stream B (the stream suffering from less spectral masking) than Stream A (single-factor ANOVA yields *F*_(1,10)_ = 6.38, *p* = 0.03).

#### Cortical Responses

**Figure 3:**
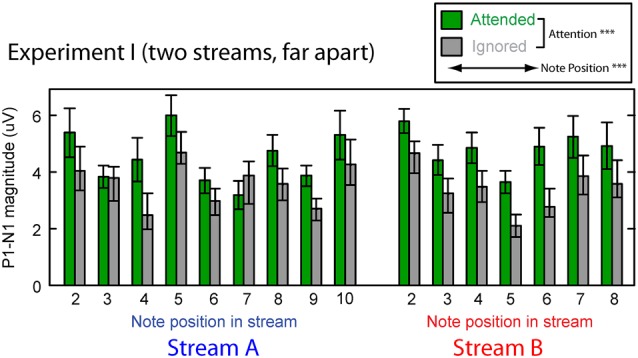
**Attentional modulation effects on the P1–N1 peak-to-peak magnitudes in Experiment I.** Each note (except the initial note) is analyzed for Stream A (left) and Stream B (right). Green bars represent response magnitudes when the corresponding stream is attended and gray bars when that stream is ignored. Error bars indicate the standard error of the mean across subjects. Main effects of both attention and note position were statistically significant (as denoted in the legend).

Figure 3 shows the averaged P1–N1 magnitudes of onset ERPs for all but the first notes in each of Stream A and Stream B. Top-down control appeared to modulate the P1-N1 magnitude, with a larger magnitude for note onsets in a stream when listeners attended to that stream compared to when they attended to the competing stream (green bars are higher than corresponding gray bars in Figure 3). The overall magnitude of the response, however, seems to vary with the temporal position of the notes in each stream.

We tested these observations using a multi-way ANOVA with main effects of attention condition (attended/ignored), temporal position of notes, and stream (A or B). There was a significant main effect of attention condition (*F*_(1,310)_ = 42.314, *p* < 0.01), confirming that attention to a stream enhances the P1-N1 response magnitude. The main effect of temporal position also was significant (*F*_(14,310)_ = 4.462, *p* < 0.01), showing that the P1-N1 magnitude varied from note to note (perhaps due to interactions between notes in Stream A and Stream B). However, neither the main effect of auditory stream (*F*_(1,310)_ = 0.869, *p* = 0.35) nor the interaction of stream and attention condition (*F*_(1,310)_ = 6.169 × 10^−3^, *p* = 0.98) reached significance. In addition, the interaction of temporal position and attention condition did not reach significance (*F*_(14,310)_ = 0.819, *p* = 0.65; note that temporal position was a nested factor, so there was no interaction term with stream). These results suggest that the strength of attentional modulation was similar across all notes in both streams, even though the average magnitude of the ERPs varied, depending on which note in which stream was considered.

**Figure 4:**
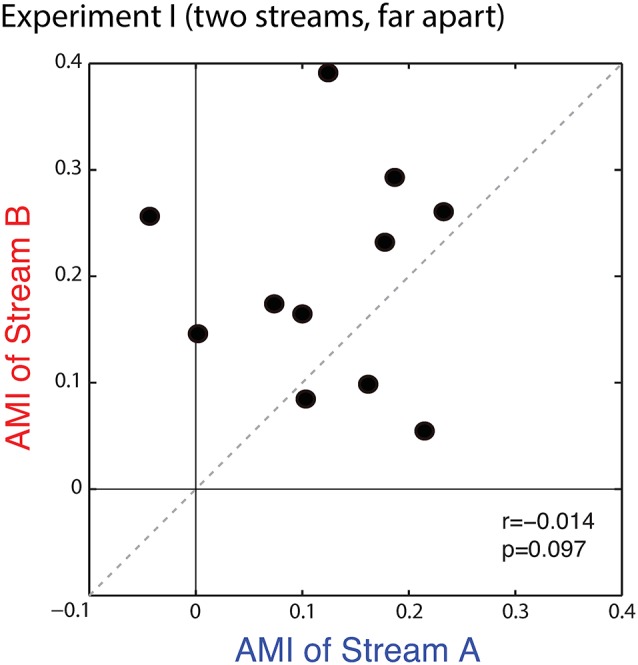
**Scatterplot of the attentional modulation index (AMI) for Stream A vs. Stream B for each subject in Experiment I.** Values generally fall in the top right quadrant (positive for both streams); however, the AMI for individual subjects is not correlated across conditions.

Figure 4 shows a scatterplot of the AMIs for Stream A vs. Stream B for each subject. All but one of the points falls in the upper right quadrant (positive AMI for both streams), confirming that the AMI tends to be positive (ERPs to a particular note are larger when is attended vs. when it is ignored). Statistical analysis confirms that at the group level, the AMI is significantly greater than zero for both Stream A and Stream B (Wilcoxon signed rank test, *p* < 0.01, signed rank = 2 for Stream A and 0 for Stream B). AMIs were similar for Stream A (mean = 0.121, standard deviation = 0.086) and Stream B (mean = 0.196, standard deviation = 0.101); repeated-measures ANOVA finds no effect of condition (*F*_(1,10)_ = 3.420, *p* = 0.09). While the individual variation in the AMI is large, individual subject AMIs for Stream A and Stream B are not correlated (*r* = −0.014, *p* = 0.97), showing that the inter-subject differences in the strength of attentional modulation are not very consistent in this task, with the rank ordering of the AMI across subjects varying for trials where listeners are attending to Stream A compared to trials where they are attending to Stream B.

#### Envelope Following Responses

We compared the EFR strength in response to tones when the stream they were in was attended and when it was ignored. For standard notes in Stream A, the PLV varied from 0.095 to 0.325 (mean of 0.173) when Stream A was attended to, and from 0.094 to 0.298 (mean of 0.165) when Stream A was ignored. For standard notes in Stream B, the PLVs varied from 0.108 to 0.495 (mean of 0.188) when Stream B was attended, and from 0.115 to 0.539 (mean of 0.193) when Stream B was ignored. There was no significant main effect of attention for either Stream A (ANOVA: *F*_(1,10)_ = 1.325, *p* = 0.28) or Stream B (ANOVA: *F*_(1,10)_ = 0.572, *p* = 0.47).

**Figure 5:**
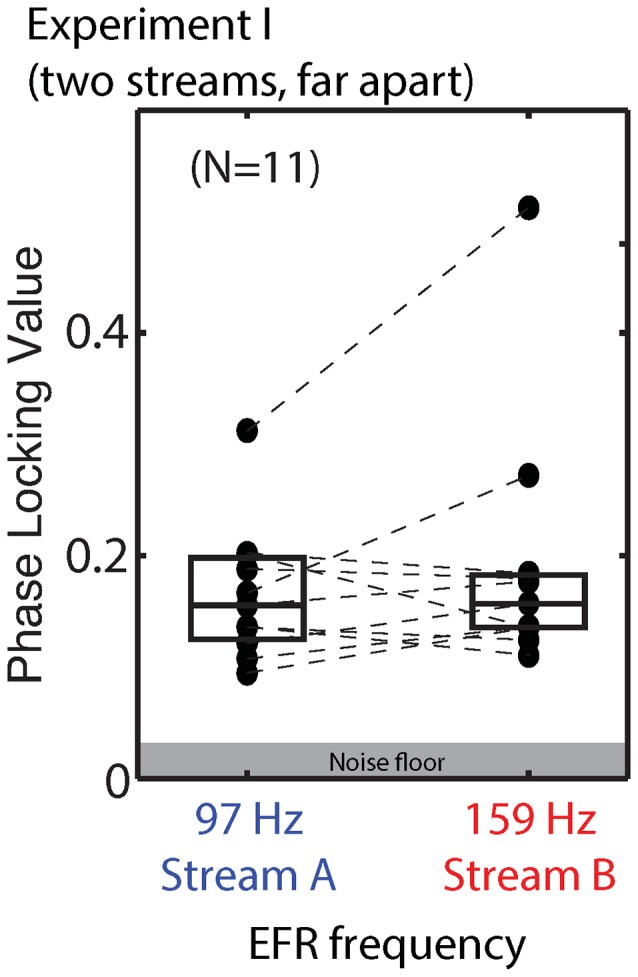
**Phase locking values (PLVs) of brainstem envelope following responses (EFRs) from standard tones in Stream A, which was spectrally fully masked (left), and Stream B, which was only partially masked (right) in Experiment I.** Connected points show results for individual subjects. Box plots denote the median (center), 25th and 75th percentiles.

Raw EFRs from the standard notes in Streams A and B are shown in Figure 5. The PLVs are relatively low for most listeners (most falling below 0.2), and on average are similar in magnitude for Stream A (97 Hz) and Stream B (159 Hz). Individual variability is large, consistent with past reports (Ruggles et al., 2012; Bharadwaj et al., 2015). For most subjects, the PLVs for the (spectrally masked) standard notes in Stream A are similar to the PLVs for the (not fully masked) standard notes in Stream B.

#### Relationships Between Behavior and Physiological Measures

**Figure 6:**
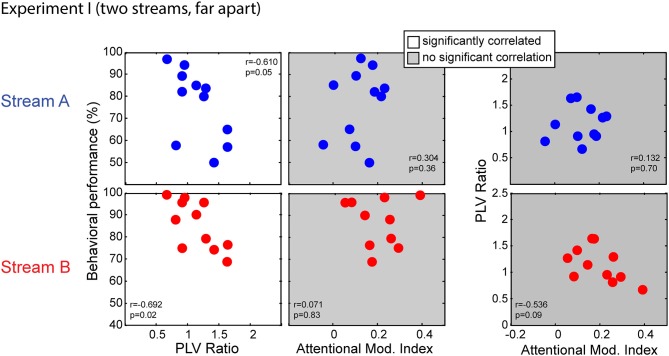
**Scatterplots of individual subject results in Experiment I for notes in Stream A (top) and Stream B (bottom).** White panels show pairs of measurements for which individual results show a significant correlation, while gray panels show measurement pairs for which there is not a statistically significant correlation. For both streams, the PLV ratio correlates with the behavioral performance but the AMI does not. There is no significant correlation between the PLV ratio and the AMI.

Simultaneous measurements of behavior scores, EFRs and ERPs allowed us to assess if behavioral performance was related to the fidelity of subcortical coding or/and attention modulation efficacy. To quantify subcortical coding fidelity, we used the PLV ratio; as discussed above, we expected smaller PLV ratios in subjects who had more robust coding fidelity, since small ratios arise when the EFR to the masked standard notes from Stream A are more similar in size to the EFR in response to the notes from Stream B. We used the AMI to quantify the strength of top-down attentional control. Figure 6 compares behavioral performance to the PLV ratio (first column) and the AMI (second column) separately for Stream A (top row) and Stream B (bottom row); the third column directly compares the PLV Ratio to the AMI for Stream A and Stream B.

Performance for both Stream A and Stream B was significantly correlated with the PLV ratio (*r* = −0.610, *p* = 0.05 for Stream A and *r* = −0.692, *p* = 0.02 for Stream B). Thus, listener performance was related to how well the brainstem encoded temporal features. In contrast, behavioral performance was unrelated to attentional modulation of cortical responses (*r* = 0.304, *p* = 0.36 for Stream A and *r* = 0.071, *p* = 0.83 for Stream B). In addition, the PLV Ratio was unrelated to the AMI (*r* = 0.132, *p* = 0.70 for Stream A and *r* = −0.536, *p* = 0.09 for Stream B).

We used a multi-regression linear model to fit the behavior data using the PLV ratio and AMI. Regression models for performance on Stream A and on Stream B were both significant (*F*_(2,8)_ = 4.379, *p* = 0.05, *R*^2^ = 0.523, betas: intercept AMI PLVR = (105.19 73.45 −32.65) for Stream A; *F*_(2,8)_ = 6.106, *p* = 0.02, *R*^2^ = 0.604, betas: intercept AMI PLVR = (129.82 −45.69 −30.74) for Stream B). We tested model fitting by dropping either the PLV ratio or the AMI (as described in Pinheiro and Bates, 2006). Dropping the PLV ratio resulted in a significantly worse fit (with a larger AIC value) to the results (*F*_(1,8)_ = 7.212, *p* = 0.03 for Stream A; *F*_(1,8)_ = 12.108, *p* < 0.01 for Stream B), whereas dropping the AMI did not (*F*_(1,8)_ = 2.520, *p* = 0.15 for Stream A; *F*_(1,8)_ = 2.538, *p* = 0.15 for Stream B). Thus, the most parsimonious model included the PLVR as a regressor, but not the AMI.

### Discussion

#### Listeners Engaged Attention During the Task, But it was not What Determined Performance

In some ways, selective focus of attention may not have been absolutely critical for performing the task of counting the number of pitch deviants contained in the target stream. Because there were never any deviants in the distracting stream, listeners could have adopted a strategy of just counting any deviant they heard. However, the pitch deviations that they had to detect were small, on the order of a third of a semitone. If listeners had been able to listen holistically to both streams at once, these small changes would likely have been hard to detect in the context of the large pitch range of the two streams, which were over eight semitones apart (e.g., see Bregman, 1990). Thus, we expected listeners to engage selective auditory attention during the task.

The data show that selective attention is deployed. The AMI, which measures how strongly the neural representation is modulated by the focus of attention, was positive both when listeners attended to Stream A and when they attended to Stream B. In other words, neural responses depended on what stream the listener was attending. It is worth noting that the strength of the neural response to individual notes varied significantly throughout the note sequences; however, these differences were likely due to systematic interactions of the overlapping neural responses to the notes making up the two streams. Consistent with this view, the degree to which attention modulated the responses to individual notes was the same across notes, whether they were in Stream A or in Stream B.

While selective attention was deployed during the task, there was no systematic relationship between the strength of attentional modulation of notes in a stream and how well listeners performed when attending that stream. Similarly, across individual subjects there was no consistent relationship in the strength of the AMI; intra-subject differences in the strength of attentional modulation to notes in Stream A and Stream B were similar to intra-subject differences in attentional modulation. These results contrast with previous studies from our lab in which individual differences in the strength of attentional modulation were related to performance and were consistent across various stimulus conditions (Choi et al., 2014).

We believe one key difference between the previous task and the current task is that it was hard to segregate the competing melodic streams in the prior study. Here, we used only two, rather than three competing streams; moreover, the spatial separation here was large (1352 μs difference between the two streams’ ITDs), whereas it was very modest in the prior experiment (100 μs difference in ITD between the adjacent streams). Because there were only two streams that were far apart, it was likely easy to direct attention to the desired stream here; listeners may have had to expend less effort to achieve suppression of the distracting stream than in the previous study.

Another key difference between the current results and those of the previous task is that the other study required listeners to judge the direction of pitch changes within the target stream while ignoring ongoing pitch changes in the distracting streams. Here, as noted above, the listeners only had to count deviants; there were no pitch variations in the distracting stream. Novel and unexpected events are salient compared to expected events; they tend to draw attention exogenously and produce bigger-than-normal neural responses. By including unpredictable deviants note patterns in the distractor, the previous task made it harder to maintain focus on the ongoing target stream. Top-down control of attention seems to have directly determined performance in that task. In turn, listeners seem to have focused attention as “strongly” as they could to perform as well as possible. Because attentional focus was not critical here, the strength of the neural modulation appears to be noisy, rather than reflecting differences in ability. This idea is consistent with the fact that although the AMI was generally positive, inter-subject differences in the AMI were not robust, differing between trials in which listeners attended to Stream A vs. trials in which they attended to Stream B.

#### Attention did not Affect the Envelope Following Response

Some studies suggest that auditory brainstem responses are modulated by attention (Hackley et al., 1990; Galbraith and Arroyo, 1993; Galbraith et al., 2003; Lehmann and Schönwiesner, 2014; Coffey et al., 2016). Similarly, a recently published study concluded that top-down control influences the distortion product otoacoustic emission (DPOAE; Wittekindt et al., 2014). However, in our lab, we have not found a significant effect of selective auditory attention on the EFR; indeed, using a Bayesian analysis, we previously concluded that any attentional effects on the EFR must be small in magnitude (Varghese et al., 2015). In reviewing other studies, we see some evidence that attention to *non-auditory* stimuli can affect EFRs in response to sound, but no strong evidence that the EFR strength to a particular note depends on whether listeners are attending to it or ignoring it in favor of a different sound (Varghese et al., 2015).

Consistent with this view, here we found no significant effects of attention on the strength of the EFR for either stream. This negative result should not be taken as proof that neural responses in the brainstem are unaffected by top-down signals related to attention. Instead, this lack of effect only allows us to conclude that the EFR metric we use (a coarse measure that depends upon a sum of an enormous number of distant electrical sources in the brain) is not sensitive to any effects of attentional modulation that may be present in the brainstem.

#### Individual Differences in Sensory Coding Determined Performance

While attentional modulation was similar for the two streams, performance was better for Stream B than for Stream A. By design, roughly half of its components in the notes of Stream B were not masked by Stream A, while all but the fundamental of the notes in Stream A overlapped in spectrum with Stream B. In other words, performance was better for the less-masked stream than the stream that was more fully masked. In addition, for both Stream A and Stream B, an individual’s performance was inversely proportional to the PLV ratio, which is a measure that should reflect differences in the strength of subcortical coding. Unlike some past studies, even though attention modulated the representations of Stream A and Stream B, the strength of this cortical effect was unrelated to performance. Together, all of these results support the idea that individual differences in the fidelity of the low-level sensory representation limited performance on this task. Indeed, the individual differences that we observe may well reflect differences in the number of auditory nerve fibers and the degree of hidden hearing loss, similar to what we have observed in a number of other recent studies of individual differences in hearing ability amongst listeners with NHTs (Ruggles et al., 2011, 2012; Bharadwaj et al., 2014, 2015).

#### Brainstem Responses were not Particularly Strong for These Stimuli

There were systematic relationships between the PLV ratio to notes in a stream and how well listeners performed when counting pitch deviants in that stream. Despite this, the PLVs that we measured were not particularly strong compared to other studies. Looking at the stimuli themselves, this is not particularly surprising. The stimulus parameters were not optimized to elicit a strong EFR: each epoch was very brief, reducing the SNR in the computed EFRs. Noise in the EFR measurement tends to be inversely proportional to frequency, and we chose rather low fundamental frequencies for the notes we used to elicit EFRs. Even so, the PLV ratio successfully captures individual differences in sensory coding strength. To enhance sensitivity to these individual differences, in Experiment II we changed the stimuli to try to improve the SNR in the EFRs we measured.

## Experiment II

Experiment II was designed to be similar in structure to Experiment I, but to reveal individual differences in the ability to focus attention. To achieve this, we adapted a “melody contour” identification task that we have previously used to study selective auditory attention (see Choi et al., 2014; the current task is described fully below). As discussed above, this previous task produced consistent individual differences in the ability to focus selective auditory attention, and these differences were related to how well listeners could identify the melody contour of the target stream.

Some key differences between Experiment I and this adapted task are summarized here. We made it harder to focus attention on the stream of interest by: (1) creating a sound mixture of three competing streams rather than two by adding a distractor (Stream C) that was presented diotically; (2) decreasing the ITDs used to lateralize Stream A and Stream B, so that the spatial cues defining the target were more subtle than in Experiment I; and (3) having each of the competing streams contain changes in pitch, rather than having pitch changes only occur in the target stream, to ensure that listeners had to concentrate on important pitch changes and block out unimportant pitch changes. All of these changes increased the need to focus attention on the target in order to make sense of its content.

We made some other changes to increase the SNR in the measured EFRs. In particular, we increased the duration of individual notes in the streams and increased the F0 of the notes used to measure EFRs. By using longer analysis epochs, each note included more cycles of the fundamental frequency, producing a less noisy estimate of the brainstem response phase. Increasing the F0 also should also increase the SNR of the EFR. Given that we did not find any effects of attentional modulation on EFRs in Experiment I (consistent with previous reports; e.g., Varghese et al., 2015), we did not design Experiment II to test for any such effect.

### Materials and Methods

#### Subjects

Sixteen subjects were recruited. One failed the screening (see below); the other fifteen (9 males, 6 females, aged 18–35) completed the experiment.

#### Stimuli

**Figure 7:**
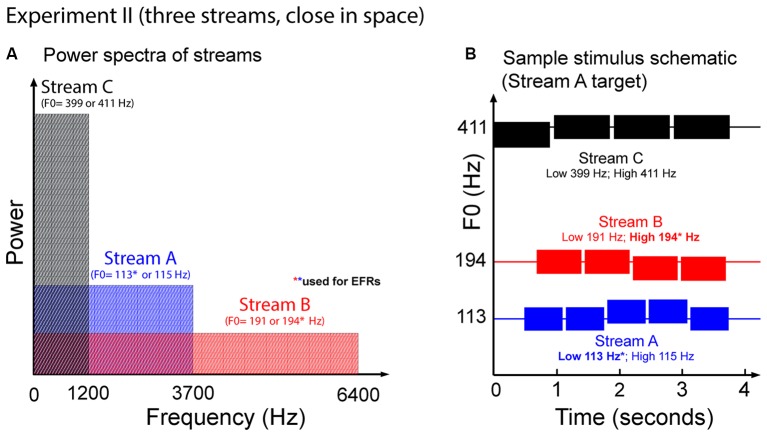
**(A)** Power spectrum of the auditory streams in Experiment II. Tones in Stream A were wholly masked by tones in Stream B, but not vice versa. **(B)** Sample schematic of auditory stimuli in Experiment II. In addition to two potential target streams, there is a third Stream C that is always a distractor. All three streams comprise simple two-note melodies. Listeners must identify the shape of the melody contour of the target stream.

Figure 7 illustrates the auditory stimuli used in Experiment II. Each trial presented potential target Streams A and B, and a distractor, Stream C. Stream C always started first. This distractor stream was made up of a sequence of four tones each of duration 939 ms, separated by an ISI of 959 ms. Stream A, which started 490 ms after Stream C, contained five tones of duration 644 ms, separated by an ISI of 664 ms. Stream B started 200 ms after Stream A, and contained four complex tones of duration 748 ms, separated by ISI of 768 ms. We used longer note durations here in order to increase the SNR of the EFRs compared to in Experiment I.

All tones in all streams were gated on and off with cosine-squared ramps (onsets: 10-ms duration; offsets: 100-ms duration) to reduce spectral splatter. Longer offset ramps were used (compared to Experiment I) to minimize offset ERPs that could interfere with the onset ERPs to subsequent notes, which can otherwise be prominent for longer-duration notes like those used here.

Each of the three streams formed simple two-note melodies of low (L) and high (H) notes. The L and H fundamental frequencies differed for the three streams, and were set to 113 Hz and 115 Hz for Stream A, 191 Hz and 194 Hz for Stream B, and 399 Hz and 411 Hz for Stream C. These differences correspond to pitch shifts of 0.3 semitones for Streams A and B, and 0.5 semitones for Stream C. On each trial, each of the streams was randomly chosen to have a melody contour that was ascending, descending, or zigzagging, with equal likelihood (1/3 each). The contours of the three melodies were chosen independently within each trial. If the contour of a given stream was ascending, it started with an L note; if it was descending, it started with an H note, and if it was zigzagging, it could start with either an L or an H note (with equal likelihood). For ascending and descending sequences, the melody changed to the other value (H or L, respectively) at some random point later in the sequence, and all subsequent notes repeated that value (e.g., valid five-note ascending sequences include LLLHH and LLLLH). For zigzagging melodies, at some point at least two notes from the end of the melody, the note value changed from the starting note value to the other value. In order to ensure that listeners had to maintain attention on the target stream throughout the sequence, zigzagging melodies always changed back to the original note value only for the final note of the melody (e.g., LLHHL, HLLLH and HHHLH are valid five-note zigzagging sequences).

Finally, EFRs were measured using the low notes in Stream A and the high notes in Stream B. In order to ensure that the melodies contained a large number of these “standard” notes, of all the possible random melodies, we biased the selection to choose relatively more Stream A melodies that had many low notes and Stream B melodies that had high notes; however, we ensured that the likelihood of each type of melody was 1/3.

Because Stream C notes only contained the lowest three harmonics of their fundamental, Stream C did not mask any frequencies above 1500 Hz (see Figure 7A). As a result, as in Experiment I, the mid and upper harmonics of the notes in Stream B were not masked by any of the other sounds in the mixture, but the mid and upper harmonics of the notes in Stream A were masked by the spectral content of notes in Stream B.

Stream C was always played diotically, so that it appeared to come from midline. Stream A and Stream B were simulated from different hemifields using rather modest ITDs of ±143 μs. Which stream was in which hemifield was chosen randomly on each trial.

#### Task Design

Each subject first performed training runs to teach them how to name “ascending, ” “descending, ” and “zigzagging” melodies. Each of the training runs consisted of 12 trials. Each training run presented a single stream (either a four-note example of Stream A or a five-note example of Stream B, chosen randomly from trial to trial). Subjects were asked to indicate the perceived melody contour using number keys: 1 for ascending, 2 for descending, and 3 for zigzagging. After the response period, the fixation dot changed color to indicate whether the reported contour was correct (blue dot) or not (red dot). Each subject performed training runs until they achieved 11 correct responses in a 12-trial run. One of the subjects failed to achieve this criterion by the end of nine training runs and was excused from the study. The remaining 15 subjects achieved the criterion after 1–7 runs (mean 2.53 runs, standard deviation 1.8 runs).

Following training, each subject performed test blocks of 40 trials each in which Streams A, B and C were presented simultaneously. Within each block, Stream A was the target stream for 20 trials and Stream B was the target stream in the other 20 trials. Although Stream C always came from in front, and thus never served as the target, subjects were never explicitly informed of this detail. Trials were presented in a different random order for each subject. Each subject performed 12 blocks for a total of 480 trials (240 with Stream A as the target and 240 with Stream B as the target). As in the training runs, subjects used number keys to indicate the target contour and received feedback after the response period.

### Data Analyses

#### Behavior

Percent correct responses were calculated separately when the target stream was Stream A and when it was Stream B. These values were computed independently for each subject, averaging across trials in the test blocks.

#### ERP Measurement

We computed $M_{\text{s},\text{n}}^{\text{Attended}}$ and $M_{\text{s},\text{n}}^{\text{Ignored}}$ for notes 2–5 (Stream A) and notes 2–4 (Stream B) for each subject. We averaged these magnitudes over notes to summarize the strength of the attentional modulation for each subject (*AMI_s_*).

#### Subcortical Measurement

As described above, we biased our selection of melodies to ensure we had a sufficiently large number of “standard” notes from which to estimate the EFR for each subject. Across all trials, the mean number of low notes in Stream A melodies was 3.0 (average of 1459 high notes or roughly 730 high notes in each polarity for each subject), while the mean number of high notes in Stream B was 2.4 (average of 1133 high notes or roughly 566 high notes in each polarity for each subject). Each of these notes was treated as a separate epoch; we computed PLVs from these distributions.

### Results

#### Behavior

The mean percentage of correct responses was 81.19% when listeners attended to Stream A (45.83–97.92%; standard deviation: 13.68%) and 82.92% when they attended to Stream B (range: 47.08–97.92%; standard deviation: 14.88%). Although individual differences were large, there was not a significant main effect of attention condition (*F*_(1,14)_ = 1.45, *p* = 0.25).

#### Cortical Responses

**Figure 8:**
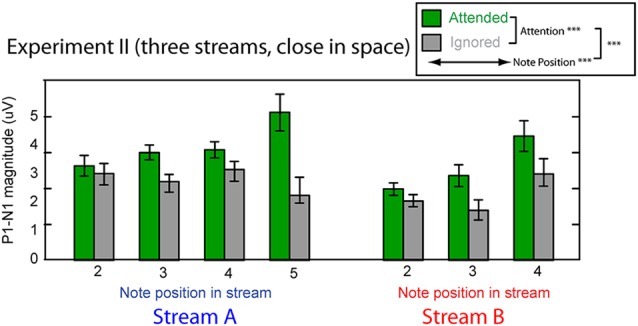
**Attentional modulation effects on the P1–N1 peak-to-peak magnitudes in Experiment II.** Each note (except the initial note) is analyzed for Stream A (left) and Stream B (right). Green bars represent response magnitudes when the corresponding stream is attended and gray bars when that stream is ignored. Error bars indicate the standard error of the mean across subjects. Main effects of attention and note position were statistically significant, as was there interaction (as denoted in the legend).

Figure 8 shows the averaged P1–N1 magnitudes of onset ERPs for all but the first notes in each of Stream A and Stream B. As in Experiment I, top-down control modulated the P1-N1 magnitude, with a larger magnitude for note onsets in a stream when listeners attended to that stream compared to when they attended to the competing stream (green bars are higher than corresponding gray bars in Figure 8). Also as in Experiment I, the overall magnitude of the response varied with the temporal position of the notes in each stream. Unlike in Experiment I, the strength of attentional modulation seemed to be larger for the final note in Stream A than for the other notes. These observations were supported by a multi-way ANOVA with main effects of attention condition (attended/ignored), temporal position of notes, and stream (A or B). As in Experiment I, there were significant main effects of both attention condition (*F*_(1,182)_ = 35.039, *p* < 0.01) and temporal position (*F*_(5,182)_ = 4.560, *p* < 0.01). In addition, however, there was a significant interaction between attention and temporal position (*F*_(5,182)_ = 3.606, *p* < 0.01). *Post hoc* interaction analyses confirmed that this interaction arose because attention had a larger effect on the last tone of Stream A than all other notes except for the last tone of Stream B2 (chi square test; *p*-value adjustment method: Holm’s method; alpha level at 0.05). Neither the main effect of auditory stream (*F*_(1,182)_ = 2.128, *p* = 0.15) nor the interaction of stream and attention condition (*F*_(1,182)_ = 2.176, *p* = 0.14) reached significance. These results suggest that the strength of attentional modulation was similar across all notes in both streams, with the exception of the final note in each stream, where attentional modulation tended to be stronger.

**Figure 9:**
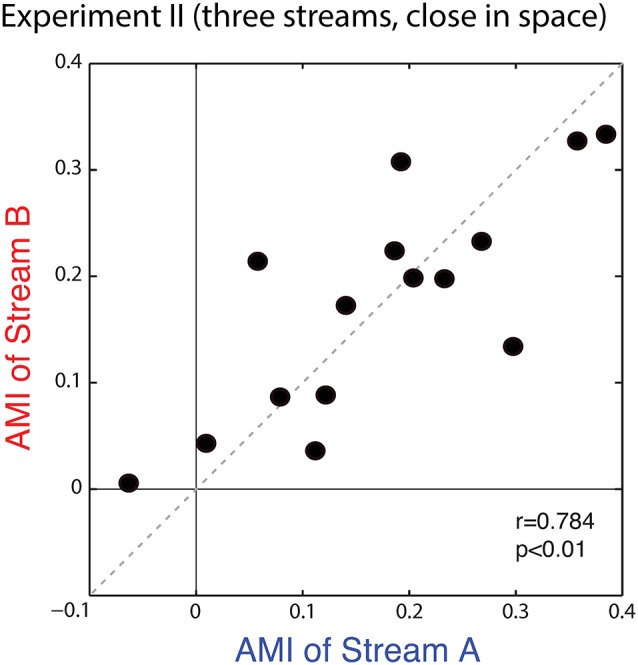
**Scatterplot of the AMI for Stream A vs. Stream B for each subject in Experiment II.** Values generally fall in the top right quadrant (positive for both streams). Moreover, across subjects, the AMI for Stream A and the AMI for Stream B are strongly correlated.

Figure 9 plots the average AMIs for Stream A vs. Stream B for each subject. As in Experiment I, the points almost all fall in the upper right quadrant (positive AMI for both streams); the AMI is significantly greater than zero for both Stream A and Stream B (Wilcoxon signed rank test, *p* < 0.01, signed rank = 3 for Stream A and 0 for Stream B). At the group level, AMIs were similar for Stream A (mean = 0.172, standard deviation = 0.126) and Stream B (mean = 0.174, standard deviation = 0.106); repeated-measures ANOVA finds no effect of condition (*F*_(1,14)_ = 0.005, *p* = 0.95). Unlike in Experiment I, here, the AMI is strongly correlated for Stream A and Stream B (*r* = 0.784, *p* < 0.01), showing that the inter-subject differences in attentional modulation are consistent. Indeed, data in Figure 9 fall along the diagonal line, showing that the strength of attentional modulation is equal for Stream A and Stream B on an individual basis, and that individual differences are large compared to the differences between the strength of attentional modulation for notes in Stream A and notes in Stream B.

#### Envelope Following Responses

**Figure 10:**
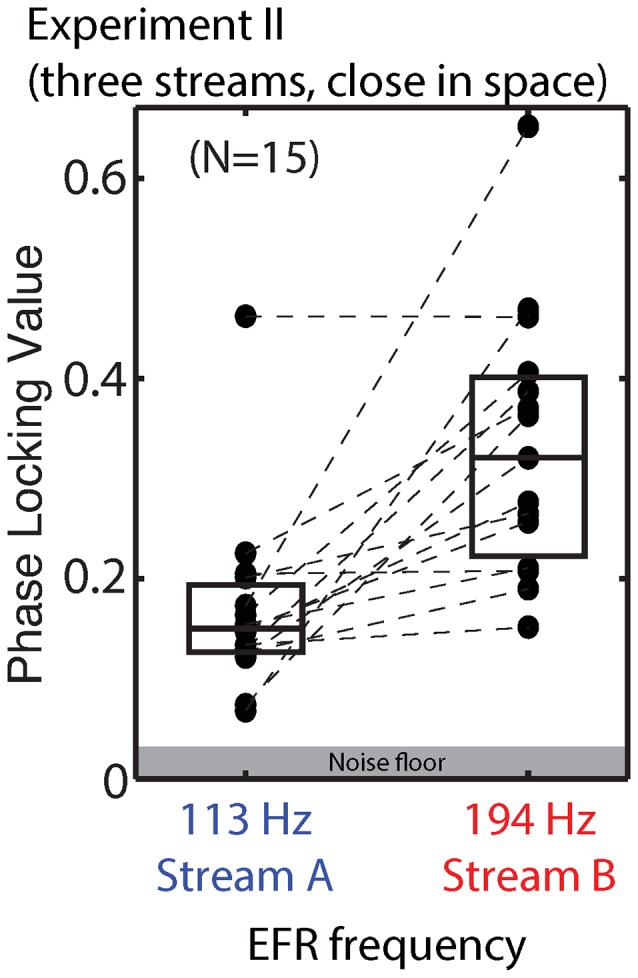
**PLVs of brainstem EFRs from standard tones in Stream A, which was spectrally fully masked (left), and Stream B, which was only partially masked (right) in Experiment II.** Connected points show results for individual subjects. Box plots denote the median (center), 25th and 75th percentiles.

We did not design the stimuli in Experiment II to allow a direct evaluation of the effect of top-down attention on the EFR, as discussed above. Raw EFRs from the standard notes in Streams A and B are shown in Figure 10. The PLVs are larger than in Experiment I, consistent with the changes in the stimuli (longer note, higher F0s). For the majority of subjects, the magnitude of the EFR to notes in Stream A is smaller than the magnitude of the EFR to the notes in Stream B.

#### Relationships Between Behavior and Physiological Measures

**Figure 11:**
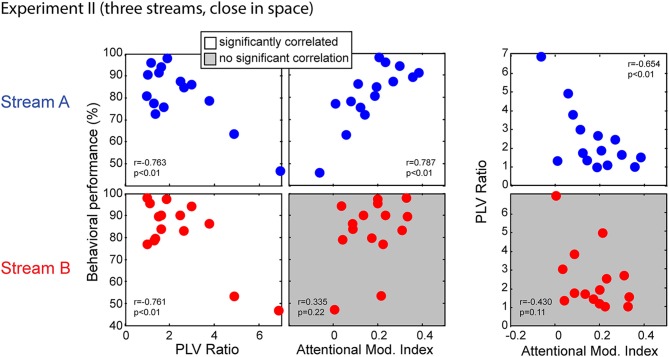
**Scatterplots of individual subject results in Experiment II for notes in Stream A (top) and Stream B (bottom).** White panels show pairs of measurements for which individual results show a significant correlation, while gray panels show measurement pairs for which there is not a statistically significant correlation. For both streams, the PLV ratio correlates with behavioral performance. For Stream A, performance is significantly correlated with the AMI, but not for Stream B. Similarly, for Stream A, the PLV ratio is significantly correlated with the AMI, but not for Stream B.

Figure 11 shows the correlations between behavior performance, AMI and PLV ratio between Stream A and Stream B in Experiment II. Performance for both Stream A and Stream B was significantly correlated with the PLV ratio (*r* = −0.763, *p* < 0.01 for Stream A and *r* = −0.761, *p* < 0.01 for Stream B). Thus, listener performance was related to how well the brainstem encoded temporal features, just as in Experiment I. It is worth noting, however, that there are two “bad” subjects with very large PLV ratios and with relatively poor performance, and that these two data points contribute strongly to this significant correlation. When these data points are left out, the correlation does not reach significance; still, these results suggest that for listeners who have very poor subcortical coding, performance on a selective attention task can fail. Unlike in Experiment I, behavioral performance for Stream A was significantly correlated with attentional modulation of cortical responses (*r* = 0.787, *p* < 0.01); moreover, the PLV Ratio and AMI for Stream A were also significantly correlated (*r* = −0.654, *p* < 0.01). This was not the case for the higher-pitched stream Stream B; performance was not correlated with attentional modulation (*r* = 0.335, *p* = 0.22) and the PLV ratio was not significantly correlated with the AMI (*r* = −0.430, *p* = 0.11).

We applied multiple linear regression models to further investigate these relationships. A model of behavioral performance including both the AMI and the PLV ratio accounted for significant variance in individual ability (*F*_(2,12)_ = 16.050, *p* < 0.01, *R*^2^ = 0.728, betas: [intercept AMI PLVR] = [80.39 54.89 −3.57] for Stream A; *F*_(2,12)_ = 8.240, *p* < 0.01, *R*^2^ = 0.579, betas: [intercept AMI PLVR] = [99.03 1.44 −6.77] for Stream B). For both Stream A and Stream B, a model that removed the dependence on subcortical coding fidelity (by dropping the PLV ratio term) resulted in a significantly worse fit (with larger AIC) to the results (*F*_(1,12)_ = 6.409, *p* = 0.03 for Stream A; *F*_(1,12)_ = 13.275, *p* < 0.01 for Stream B). This confirms that, for both streams, accounting for the fidelity of peripheral coding improved predictions of how well a listener performed on the selective attention task. In contrast, performance when listeners attended to Stream B was fit equally well by the full model and a model that removed the dependence on attentional modulation (dropping the AMI term; *F*_(1,12)_ = 0.002, *p* = 0.96). Thus, the most parsimonious model of individual performance for Stream B, whose high-frequency content was not masked, depends only on sensory coding fidelity in the brainstem. In contrast, when considering performance when listeners attended to Stream A, the full model was significantly better (with smaller AIC) than the model that did not include the AMI (*F*_(1,12)_ = 4.755, *p* = 0.05). This confirms that the strength of attentional modulation accounts for differences in individual performance above and beyond the variance accounted for the fidelity of subcortical coding.

### Discussion

#### Individual Differences in Sensory Coding are Related to Task Performance

Like in Experiment I, individual differences in performance for both streams were correlated with the strength of the brainstem response (as summarized by the PLV ratio). Given that in both experiments, listeners had to process small pitch differences in notes within the target stream, this dependence makes sense. Unlike in Experiment I, in Experiment II performance was equally good whether listeners attended to the stream whose spectrum was fully masked (Stream A) or the stream whose upper harmonics were not masked (Stream B). Given that the amount of peripheral masking for the two streams differs, the fact that performance is equally good suggests that, although individual differences in coding fidelity impacted performance, they were not the only factor influencing individual performance.

#### Attention Affects Cortical Coding Similarly Strongly and Similarly for the Two Streams

Just as in Experiment I, we found strong attentional modulation of both Stream A and Stream B, confirming that listeners deployed selective attention to perform the task. On a group level, the strength of attentional modulation was the same for Stream A and Stream B. More importantly, on an individual basis, the strength of attentional modulation was essentially equal for the two streams.

Listeners were cued as to which direction the target stream would come from, but until the streams began to play, they did not know whether the stream in the target direction was Stream A or Stream B. Thus, it makes sense that the top-down effects of attention that allowed listeners to focus on the target stream at its start were similar for Stream A and Stream B; this helps explain why the physiological effects of attention on cortical coding was equal for the two streams.

In Experiment I, the strength of attentional modulation on Stream A and Stream B was not consistent for individual subjects, even though there was no difference between the strength of attentional modulation at a group level. However, in Experiment I, attentional modulation was not as critical for performing the task: only the target contained deviants, there were only two streams, and the streams had very different ITDs. The consistency of the inter-subject differences in attentional modulation in Experiment II suggests that listeners did their best to attend to the target source and filter out the competing streams here, producing more consistent attentional modulation of cortical responses, which therefore more consistently reflected an individual’s ability to control top-down attention.

#### Top-Down Attention Effects are Related to Performance, at Least for the Masked Stream

For Stream A (the masked stream), the strength of attentional modulation correlated with both performance and with the strength of the subcortical coding fidelity. Neither of these relationships was significant for Stream B.

At first glance, these results seem to suggest that for the stream that was spectrally masked (Stream A), the efficacy of attentional modulation is determined by the fidelity of sensory coding. For the fully masked Stream A (but not the only partially masked Stream B), the fidelity of peripheral coding might determine how well the stream could be segregated from the sound mixture. Especially for the listeners with a weak sensory representation, segregation might fail, which would in turn interfere with the ability to modulate sensory responses based on attention. Yet, if this were the full explanation, one might expect the strength of attentional modulation to be stronger for Stream B than Stream A. Instead, the AMI was equal for the two streams. Moreover, our regression analysis shows that for fully masked Stream A, both the peripheral coding strength and the strength of attentional modulation contribute independently to fitting performance. Thus, the individual differences in attentional modulation for Stream A are affected by peripheral coding fidelity, but there are additional, more central individual differences that also affect performance.

Top-down attention is equally strong for both streams. Yet, while the efficacy of top-down attention affects performance for Stream A, we do not see a statistically significant relationship between top-down attentional modulation and performance for Stream B. One might try to reason that this is because Stream B, whose upper harmonics are not masked, is easier to analyze than the masked stream, even when top-down attention fails. Of course, if this were the right explanation, one would expect performance to be better overall for Stream B than for Stream A, which was not the case.

Ultimately, the failure to see a relationship between the strength of attentional modulation and performance for Stream B on an individual level may simply be due to a lack of statistical power. Indeed, the AMI for Stream B is estimated from only three notes, rather than four (for Stream A). We see significant correlations between all pairs of performance, the PLV ratio, and the AMI for Stream A. For Stream B, we find only a significant correlation between performance and the PLV ratio; we do not find any relationship between the AMI and either performance or the PLV ratio. Thus, estimates of the strength of the AMI for Stream B may be noisy at the level of the individual subject.

Still, compared to Experiment I, in Experiment II individual differences in the strength of top-down attention are more consistent (correlated significantly for Stream A and Stream B). Furthermore, at least for Stream A, there is a significant relationship between the physiological measure of top-down attentional strength and performance.

#### The Strength of Attentional Modulation is Greater for the Final Note than Earlier Notes

The strength of attentional modulation varied significantly with the temporal position of a note in the stream in Experiment II. *Post hoc* analysis showed that attention had a stronger effect on the final note of Stream A than on all other notes except for the final note of Stream B. In Experiment I, although the strength of the neural response varied from note to note, all notes were equally affected by attentional focus.

One key factor for this difference may be in the design of Experiment II. In this experiment, we ensured that listeners had to maintain focus to the end of each stream by guaranteeing that, when a stream was “zigzagging,” the final change in pitch happened between the penultimate and final notes. In contrast, in Experiment I, we did not guarantee that listeners had to listen to the end of each stream in order to know the correct answer (the count of the number of deviants in the target). Moreover, because no deviants occurred in the distractor stream in Experiment I, attention was not, itself, as critical for performing the task. Given this, we may see dynamic effects of sustaining attention in Experiment II that were not evident in Experiment I.

Sustaining attention to a stream has previously been shown to improve the selectivity of auditory attention through time, both when attention is directed to spatial location, as here (Best et al., 2008, 2010), and when attention is directed to the non-spatial attribute of talker gender (Bressler et al., 2014). The current results may simply reflect the same kind of improvement in attentional focus through time; attentional focus may be growing stronger from note to note throughout the streams, but the effect may only reach statistical significance for the final note in Stream A.

However, there is an alternative explanation for the increase in the strength of attentional modulation on the final note. Because of the task design, the amount of information that listeners glean from listening to the final note of the target stream is greater than the amount of information they get from any other note in the stream. Thus, it may be that the “amount” of attention that listeners deployed varies with how important a particular note is for the task. For instance, brain activity “entrains” to ensure that coding of key events at expected times (e.g., see Riecke et al., 2015).

The current results cannot differentiate between the possibility that sustained attention to an ongoing stream grows in strength through time and the possibility that top-down attentional effort changes dynamically based on task demands. Moreover, these two possibilities are not mutually exclusive. Further experiments on the dynamics of auditory attention could help elucidate how these factors contribute to performance.

## Overall Summary

Experiment I used a simple two-source mixture of streams that were very far apart in space. Listeners had to simply count pitch deviants in a target and there were no pitch deviants in the competing stream. Even though one might not think that attention was critical for this task, the focus of attention changed the neural representation of the auditory scene, leading to weaker cortical responses to a stream when it was a distractor relative to when it was the target. Individual differences in ability were related to individual differences in subcortical coding. However, performance was not significantly correlated to the strength of attentional modulation.

Experiment II used a three-source mixture of streams that were separated by small spatial cue differences. Moreover, the distracting streams contained changes in pitch from note to note, making them salient and increasing the need to suppress them in order to make sense of the target stream. As in Experiment I, individual differences in performance were related to individual differences in subcortical coding. However, unlike in Experiment I, we found large and consistent inter-subject differences in the strength of attentional modulation, suggesting that subjects did their best to suppress the competing sounds, rather than only loosely or inconsistently focusing attention. Moreover, how well listeners were able to identify a target melody contour was significantly correlated with how strongly attention modulated the cortical representation of the scene, at least for the stream that was spectrally masked.

Together, these results show that in complex listening situations, where there are distracting sound sources, individual differences arise from multiple sources. Individual differences in sensory coding can contribute to differences in ability, even amongst listeners with NHTs. Such differences are consistent with hidden hearing loss: they manifest not just in differences in perceptual ability, but also in differences in the robustness of physiological measures of subcortical neural responses. On the other hand, when top-down attention is important to filter out distracting sounds from a mixture, the efficacy of top-down attention also influences performance.

## Author Contributions

LD and BGS-C conceived of and designed the experiment, and prepared the manuscript. LD collected and analyzed the data.

## Conflict of Interest Statement

The authors declare that the research was conducted in the absence of any commercial or financial relationships that could be construed as a potential conflict of interest.
